# Combinatorial Therapy of Letrozole- and Quercetin-Loaded Spanlastics for Enhanced Cytotoxicity against MCF-7 Breast Cancer Cells

**DOI:** 10.3390/pharmaceutics14081727

**Published:** 2022-08-18

**Authors:** Aml I. Mekkawy, Nermin E. Eleraky, Ghareb M. Soliman, Mohamed G. Elnaggar, Marwa G. Elnaggar

**Affiliations:** 1 Department of Pharmaceutics and Clinical Pharmacy, Faculty of Pharmacy, Sohag University, Sohag 82524, Egypt; 2 Department of Pharmaceutics, Faculty of Pharmacy, Assiut University, Assiut 71526, Egypt; 3 Department of Pharmaceutics, Faculty of Pharmacy, University of Tabuk, Tabuk 71491, Saudi Arabia; 4 Department of Clinical Pathology, South Egypt Cancer Institute, Assiut University, Assiut 71526, Egypt; 5 Department of Industrial Pharmacy, Faculty of Pharmacy, Assiut University, Assiut 71526, Egypt or; 6 Department of Industrial and Physical Pharmacy, Purdue University, 575 Stadium Mall Drive, West Lafayette, IN 47907, USA

**Keywords:** breast cancer, combination therapy, letrozole, quercetin, spanlastics

## Abstract

Breast cancer is the most widespread cancer in women with rising incidence, prevalence, and mortality in developed regions. Most breast cancers (80%) are estrogen receptor–positive, indicating that disease progression could be controlled by estrogen inhibition in the breast tissue. However, drug resistance limits the benefits of this approach. Combinatorial treatment could overcome the resistance and improve the outcome of breast cancer treatment. In the current study, we prepared letrozole-(LTZSPs) and quercetin-loaded spanlastics (QuSPs) using different edge activators—Tween 80, Brij 35, and Cremophor RH40—with different concentrations. The spanlastics were evaluated for their average particles size, surface charge, and percent encapsulation efficiency. The optimized formulations were further examined using transmission electron microscopy, Fourier transform infrared spectroscopy, in vitro drug release and ex vivo skin permeation studies. The prepared spherical LTZSPs and QuSPs had average particle sizes ranged between 129–310 nm and 240–560 nm, respectively, with negative surface charge and high LTZ and Qu encapsulation (94.3–97.2% and 97.9–99.6%, respectively). The in vitro release study of LTZ and Qu from the selected formulations showed a sustained drug release for 24 h with reasonable flux and permeation through the rat skin. Further, we evaluated the in vitro cytotoxicity, cell cycle analysis, and intracellular reactive oxygen species (ROS) of the combination therapy of letrozole and quercetin either in soluble form or loaded in spanlastics against MCF-7 breast cancer cells. The LTZSPs and QuSPs combination was superior to the individual treatments and the soluble free drugs in terms of in vitro cytotoxicity, cell cycle analysis, and ROS studies. These results confirm the potential of LTZSPs and QuSPs combination for transdermal delivery of drugs for enhanced breast cancer management.

## 1. Introduction

Breast cancer, the most invasive cancer among women globally, is the primary cause of cancer-related deaths among women worldwide [1]. In case of local stage tumor, the 5-year survival rate is 99%, compared to 86% and 27% at regional stage and metastatic breast cancer, respectively [2]. Early detection through breast screening (mammogram) and advanced therapeutic strategies result in better prognosis and improved survival rates [3]. Since most breast cancers (80%) are estrogen receptor–positive (ER+) [4], the growth and survival of cancerous breast epithelial cells are promoted by estrogen through binding to ER [5]. Accordingly, the estrogen-dependent progression of most breast cancers could be disrupted by drugs interfering with estrogen binding to estrogen receptors (as tamoxifen), downregulating ER (as fulvestrant) or inhibiting estrogen production using aromatase inhibitors (as letrozole; LTZ) [6]. In postmenopausal women, tissue estrogen concentrations are superior to plasma estrogen concentration [5]. Further studies proved that almost 100% of the biologically active estrogen is produced locally [7]. Therefore, the specific inhibition of estrogen levels in the breast tissue could be more beneficial for disease management. Additionally, local estrogen inhibition could overcome the side effects reported from patients under hormonal therapy, resulting from depletion of the circulating estrogen as hot flashes [8] and bone deterioration [9]. Therefore, beyond the more patient compliance achieved with the drug transdermal delivery, it provides high drug concentration at the site of application and low plasma drug concentration with reduced systemic side effects. The transdermal patches of different aromatase inhibitors were developed for site-specific delivery in the breast cancer region to achieve high local drug concentrations [5,10,11]. Aromatase inhibitors are associated with resistance development; therefore, drug combination strategies may bypass or delay resistance and improve treatment outcome [12]. The combination of the aromatase inhibitor; LTZ with antiestrogen (fulvestrant) [13], multi-kinase inhibitor (sorafenib) [14], COX-2 inhibitor (celecoxib) [12], polyphenolic flavonoids (quercetin, Qu) [15] were previously studied and showed high and synergistic efficacy on the apoptosis and proliferation inhibition of breast cancer cells.

In the current study, the combination therapy of the aromatase inhibitor (LTZ) and polyphenolic flavonoids (Qu) will be investigated. LTZ is a nonsteroidal aromatase inhibitor that can slow down the progress of certain types of breast cancer cells by decreasing the amount of estrogen produced [16]. Qu is a plant flavonoid of polyphenols, which has shown beneficial therapeutic effects against different types of cancers as breast [17], lung [18], nasopharyngeal [19], kidney [20], colorectal [21], prostate [22], pancreatic [23], and ovarian cancer [24]. Qu acts as a free radical scavenger, inhibits signal transduction, induces cancer cell apoptosis, and inhibits tumor proliferation and metastasis [17]. Various cytotoxic mechanisms of action of Qu are crucial in its combination treatment with other drugs. LTZ and Qu combination was previously shown to inhibit cell development in MCF-7 and MDA-MB-231 cells and stimulated mitochondrial apoptosis [15], where a clear synergistic cytotoxic effect was reported in the LTZ and Qu combination in MCF-7 and MDA-MB-231 cell lines. Combinatorial treatment of Qu with estrogen-sensitive chemotherapy, such as LTZ, augments the effect of these drugs, as the antiapoptotic effect of Qu is more pronounced in cells with ER+ [25]. Moreover, LTZ and Qu combination increases the Foxo1 protein (which is reported as a tumor suppressor) and gene expression in ER+ cells, which is important for anti-cancer mechanisms activation [15,26]. Therefore, this combination has the ability to improve the clinical outcomes of breast cancer treatment.

Generally, the formulation of drugs in nanoparticulate systems results in superior delivery or uptake by target cells compared with the free drugs [27]. The use of nanoparticles for transdermal drug delivery is advantageous over conventional routes because it decreases drug loss due to the first pass effect of the liver, and delivers therapeutic drugs at a controlled amount [28]. Drug formulation in nanoparticulate systems, particularly spanlastics, significantly improves therapeutic efficacy, increases drug bioavailability, and reduces toxicity of the formulated drug [29]. Spanlastics are surfactant-based elastic deformable nanovesicles that are formed of a non-ionic surfactant and an edge activator (EA) [30]. Spanlastics are non-immunogenic, biodegradable systems that are compatible with biological membrane with minimum toxicity due to presence of non-ionic surfactant [31]. They are superior to conventional nanoparticles in transdermal drug delivery because of the presence of EA that confers the penetration enhancing effect to spanlastics that facilitate drug delivery. Spanlastics squeeze themselves through biological membrane, as skin pores in case of transdermal delivery, without being disrupted due to elastic deformable nature [32].

Given these proven benefits of spanlastics as transdermal drug delivery systems, we set about to explore their features to solubilize LTZ/Qu, control their release, and enhance their anticancer efficacy. To date, no reports are published on using spanlastics for the combined delivery of LTZ/Qu. The aim of this study is thus bi-fold; first to study the effect of encapsulating LTZ and Qu on their anti-cancer potential, and second to examine the efficacy of their combination, in comparison with individual drugs against MCF-7 cells. We hypothesize that using LTZ and Qu loaded in the same nanoparticulate system could facilitate the coordinated delivery of drug combinations with different therapeutic properties. Therefore, we developed and characterized different spanlastic formulations loaded with each drug (either LTZ or Qu) using different types and ratios of EA. After that, we evaluated the in vitro release and ex vivo permeation of LTZ/Qu from each selected LTZ-loaded spanlastics (LTZSPs)/Qu-loaded spanlastics (QuSPs) through rat skin. Then, to prove our concept, we tested the cytotoxic activity of LTZ, Qu, and their combination, compared to LTZSPs, QuSPs, and their combination against MCF-7 cell line using MTT assay. Finally, we studied their effect on MCF-7 cell cycle using flow cytometry and the intracellular reactive oxygen species (ROS) levels using enzyme-linked immunosorbent assay (ELISA).

## 2. Materials and Methods

### 2.1. Materials

Quercetin was kindly gifted by Searle, Augusta, GA, USA. Letrozole was a kind donation from PHARCO Pharmaceuticals Inc. (Alexandria, Egypt). Span 60 and Brij 35 were purchased from Sigma-Aldrich Co. (St. Louis, MO, USA). Tween 80 and ethanol were obtained from El-Nasr Pharmaceutical Chemicals Co. (Cairo, Egypt). Cremophor RH40 was purchased from CISME (EMAROL H40^TM^, Milano, Italy). All other chemicals and reagents used were of analytical grade.

### 2.2. Preparation of LTZSPs and QuSPs

Drug-loaded spanlastic dispersions were prepared using ethanol injection method, as previously reported [30] using different compositions, as shown in Table 1. Briefly, specified amounts of Span 60 and drug (either LTZ or Qu) were dissolved in 2 mL of absolute ethanol and kept at 50 °C, while EA was dissolved in 10 mL preheated double distilled water (DDW, 60 °C). The ethanolic solution was then slowly injected into the preheated aqueous solution. After 3 h of stirring to remove alcohol, the dispersion was sonicated for 2 min and stored overnight at refrigerator (~4 °C). The effects of different types of EA (Tween 80, Brij 35 and Cremophor RH40) and different Span 60: EA ratios (80:20 and 60:40) were studied. The effect of using different drug amounts was also investigated.

### 2.3. In Vitro Characterization of LTZSPs and QuSPs

#### 2.3.1. Particle Size, Polydispersity Index and Zeta-Potential Measurements

Malvern Zetasizer Nano series ZS instrument (Malvern Instruments, Malvern, UK) was used to determine the average hydrodynamic diameters, size distribution (polydispersity indices, PDIs), and zeta-potential values of the formulated LTZSPs and QuSPs dispersions in DDW at ~1 mg/mL at room temperature.

#### 2.3.2. Estimation of the Encapsulation Efficiencies of LTZSPs and QuSPs

Drug encapsulation efficiency of the prepared spanlastics was calculated by the indirect method [33]. Briefly, the freshly prepared drug-loaded spanlastics were centrifuged using Amicon^®^ Ultra-15 centrifugal filters (100 kDa NMWL, Merk Millipore, Darmstadt, Germany) at 6000 rpm for 30 min. Next, the supernatant was collected and analyzed using Shimadzu UV-Vis spectrophotometer (model UV-1601 PC, Kyoto, Japan) at λ = 240 nm or λ = 372 nm for LTZ and Qu, respectively. Finally, the drug concentration was calculated from calibration curves. Encapsulation efficiency was calculated from the following equation:

Encapsulation efficiency (%) = ((Total drug concentration − drug concentration in supernatant)/Total drug concentration) × 100(1)

#### 2.3.3. Evaluation of LTZSPs and QuSPs Morphology

The selected formulations of LTZSPs (L8) and QuSPs (Q5) were imaged using a JEOL 100 CX II TEM (transmission electron microscope, Tokyo, Japan). A drop of particle dispersion (~1 mg/mL) was placed on a carbon-coated copper grid, negatively stained with 1% phosphotungstic acid and allowed to air dry prior to imaging.

#### 2.3.4. Fourier Transform Infrared Spectroscopy (FTIR) Studies

FTIR spectra of the dried LTZSPs (L8) and QuSPs (Q5), Tween 80, pure powders of LTZ, Qu, Span 60, and Brij 35 were recorded using a Nicolet 6700 FT-IR spectrometer (Thermo Fisher Scientific, Waltham, MA, USA) in the wavenumber range of 4000 to 400 cm^−1^.

#### 2.3.5. In Vitro Release Study of the Selected LTZSPs (L8) and QuSPs (Q5)

The in vitro release of LTZ and Qu from the selected spanlastic formulations was studied, as previously reported [34,35]. One-half mL of L8 preparation (equivalent to 0.75 mg LTZ) or one mL of Q5 formulation (equivalent to one mg Qu) was placed over a previously soaked cellulose membrane (Spectra/Por^®^ dialysis membrane with molecular weight cutoff 12,000–14,000) held at the lower end of a glass cylinder. After that, the glass cylinder was immersed in a beaker, including either hydroalcoholic phosphate buffer saline (PBS) solution (60 mL, PBS pH 7.4: methanol; 1:1) or PBS pH 7.4 plus 1% Tween 80 (50 mL) for LTZSPs and QuSPs, respectively. The temperature was maintained at 37 ± 0.5 °C and the stirring rate was set to 50 rpm using a thermostatically controlled water bath (Gesellschaft für Labortechnik GmbH, Burgwedel, Germany). At predetermined time points, aliquots of 3 mL sample were withdrawn and replaced with a freshly prepared release medium. Controls containing same concentration of free drug dispersions were tested, along with the spanlastic dispersions. The drug content of the release samples was assessed via utilizing a UV-Vis spectrophotometer (Shimadzu Seisakusho, Ltd., Kyoto, Japan) at λ = 240 nm or λ = 372 nm for LTZ and Qu, respectively. The cumulative percent drug released was plotted against time. The experiment was performed in triplicate.

### 2.4. Ex Vivo Skin Permeation and Deposition Studies of LTZSPs and QuSPs

The animal experiments were authorized by the Ethical Review Board of the Faculty of Pharmacy, Assiut University, Assiut, Egypt (Ref: S25-21, December 2021). Male Sprague Dawley rats (6–8 weeks) were acquired from the University Central Animal House Facility. All animals were fed with access to drinking water that was purified. The rats were retained at a room temperature of 25 ± 5 °C.

Rats were anesthetized with chloral hydrate and shaved gently with electric clippers and skin samples were excised from their dorsal side. Skin samples were then mounted at the lower end of a glass cylinder, with the stratum corneum side facing the donor compartment [36]. The glass cylinder was then submerged in a beaker containing either hydroalcoholic PBS solution (60 mL, PBS pH 7.4: methanol; 1:1) or PBS pH 7.4 plus 1% Tween 80 (50 mL) for LTZSPs and QuSPs, respectively. The donor cell was loaded with either the optimized LTZSPs (L8, one-half mL equivalent to 0.75 mg LTZ) or QuSPs (Q5, one mL equivalent to one mg Qu), and the diffusion was compared with the controls (same drug concentration dispersed in DDW). The diffusion cells were agitated at 50 rpm for 24 h at 37 ± 0.5 °C. Three mL aliquots were withdrawn at specified time, replaced with equal volume of the fresh medium, and analyzed as previously mentioned in the in vitro release study section. The permeation profiles were constructed by plotting the cumulative amount of drug permeated per unit of skin membrane area (Q_n_, mg/cm^2^) versus time (h). Apparent permeability coefficients (*P_app_* values) were estimated according to the following equation:

(2) $$P_{app}=\Delta Q/\Delta t/\left(C_{0\;}\times A\right)$$

where Δ*Q*/Δ*t* = linear mass appearance rate of the solute of interest in the receiver compartment, *C*_0_ = initial solute concentration in the donor compartment, and *A* = surface area of the skin membrane (i.e., 4.9 cm^2^). The steady-state flux (*Jss*, mg/cm^2^·h) was calculated from the slope of the plot using linear regression analysis [37,38].

After 24 h, the skin was withdrawn from the cells and washed with PBS and DDW to remove the excess drug. The skin was sliced into small pieces and homogenized. The drug remaining in the homogenized skin (deposited inside the skin) was extracted with methanol (48 h), sonicated for 30 min, centrifuged at 10,000 rpm for 60 min, and the concentration of either LTZ or Qu in the supernatant was determined spectrophotometrically as described above [39].

### 2.5. Cell Culture and Cell Viability Assay

MCF-7 cell line was obtained from the American Type Culture Collection (ATCC, Manassas, VA, USA). MCF-7 cells were cultured in Dulbecco’s modified Eagle’s medium (DMEM, Invitrogen, Waltham, WA, USA), supplemented with 10% (*v*/*v*) fetal bovine serum (Hyclone, Logan, UT, USA), 10 µg/mL of insulin (Sigma, St. Louis, MO, USA), and 1% penicillin/streptomycin (100 U/mL, Gibco, NY, USA), and incubated at 37 °C for 24 h.

Serial dilutions (10–150 µM) of soluble LTZ, Qu (in DMSO), and combination of the two (1:1), and LTZSPs, QuSPs, and their combination (1:1) were tested for effects on the viability of MCF-7 cells. One day before treatment, cells were seeded in 96-well plates at a density of 1.2 × 10^4^ cells/well. Treatments were then added as specified and after two days, media was aspirated and replaced with 100 μL fresh media and 10 μL MTT reagent (thiazolyl blue tetrazolium bromide, catalog no. M5655, Sigma-Aldrich, St. Louis, MO, USA), and plates were incubated at 37 °C for 2 h. Absorbance was measured spectrophotometrically at λ = 570 nm. Cell viability was stated as the absorbance percentage from treated cells, compared to that from the untreated cells. IC_50_ values for LTZ, Qu and their combination and LTZSPs, QuSPs and their combination were calculated using GraphPad Prism software. Additionally, the LTZSPs and QuSPs combination was evaluated using CompuSyn software (ComboSyn Inc., Paramus, NJ, USA) and combination index (CI) values were computed, where CI < 1 indicates synergy.

### 2.6. Cell Cycle Analysis by Flow Cytometry

Flow cytometry was used for quantitative assessment of apoptosis by dual staining the cells with annexin-V/propidium iodide (PI). It is based on using annexin-V coupled with fluorescein isothiocyanate (FITC) to label phosphatidyl serine sites on the membrane surface of apoptotic cells, as well as PI to flag cellular DNA in necrotic cells when the cell membrane has been completely degraded.

MCF-7 cells were plated at a density of 1 × 10^5^ cells/well in 6-well plates and incubated overnight. Then, cells were treated with soluble LTZ, Qu (in DMSO), and combinations of the two (1:1) and also by LTZSPs, QuSPs, and their combination (1:1) and incubated for 24 h. Treatments were added at 50% of the specified IC_50_ in the aforementioned cytotoxicity study. After that, the cells were rinsed, trypsinized, and washed with DMEM medium, collected in falcon tubes in which respective supernatant was collected and cell pellets were washed twice using PBS. Then, the cell suspension (100 μL) was transferred to a FACS tube and mixed with 5 μL annexin V-FITC and 5 μL PI. Tubes were gently vortexed and incubated for 30 min at room temperature in the dark. Finally, 400 μL of the binding buffer were added to the FACS tube, and the tubes were run through a FACS machine within 1 h.

Analysis of the different population of cells was done to determine apoptosis by assessing the annexin V-FITC/PI. Viable cells that were unlabeled, early apoptotic cells bound to annexin-V FITC only, late apoptotic cells bound to both annexin-V FITC/PI, and necrotic cells bound to PI only. Moreover, cell percentages were identified in each cell cycle (G_0_/G_1_, S, and G2/M). Analysis was done using the FACSCalibur system (BD, San Jose, CA, USA). The data were analyzed with Cell Quest software (BD, San Jose, CA, USA).

### 2.7. Assessment of Intracellular Reactive Oxygen Species (ROS) by ELISA

The human breast cancer cell lines MCF-7 were seeded in DMEM supplemented with 10% *v*/*v* FBS and 1% penicillin/streptomycin in 96-well plates at a density of 1.2 × 10^4^ (37 °C, 5% CO_2_). The medium was then removed, and standard solutions and the treatments were added in 0.1 mL/well each (*n* = 3). The treatments (soluble LTZ, Qu in DMSO and their combination and LTZSPs, QuSPs and their combination) dispersed in DMEM were added at specified concentration (50% of the specified IC_50_ in the aforementioned cytotoxicity study) and left for 24 h, while control wells were left untreated. An amount of 100 μL of the detection antibody working solution were added to each well, covered, and incubated at room temperature for 2 h with shaking at 400 rpm. Then, plates were incubated at 37 °C for 90 min. The plate content was then discarded and 0.1 mL of biotin-detection antibody working solution was added into the standard, the test sample, and the control wells, and plates were incubated at 37 °C for 60 min and then washed 3 times with wash buffer. Streptavidin-HRP (SABC) working solution was added into each well, and plates were incubated again at 37 °C for 30 min and washed 5 times with wash buffer. An amount of 90 μL of TMB define substrate was added into each well and incubated at 37 °C in dark within 15–30 min. Finally, 50 μL of stop solution was added into each well and mixed thoroughly. The optical density absorbance was measured at 450 nm using a microplate reader immediately after adding the stop solution. The ROS content was determined based on the absorbance difference between the tested samples and control, and a calibration curve was drawn with standard solutions.

### 2.8. Statistical Analyses

GraphPad Prism software for Windows version 8.3.0 (GraphPad Software Inc., San Diego, CA, USA) was used for the statistical analyses. Data were analyzed using two-tailed unpaired *t*-test or one-way analysis of variance (ANOVA) followed by Tukey post-hoc test to compare the effect of different drug concentrations and different types and ratios of EA on the average particle size, the zeta potential, the drug encapsulation efficiencies of the prepared spanlastics, and the permeation study data. Moreover, the results of IC_50_ and ROS concentrations between the free drugs and their combination, and the drug-loaded spanlastics and their combination using MCF-7 cell lines were compared.

## 3. Results and Discussion

### 3.1. Preparation and Characterization of Drug-Loaded Spanlastics

LTZSPs and QuSPs were successfully prepared by ethanol injection method using different types of EAs: Tween 80 (HLB:15), Brij 35 (HLB:16.9), and Cremophor RH40 (HLB:14–16). The effect of varying the concentrations of edge activator and drugs on the spanlastics particle size, polydispersity index (PDI), zeta potential, and encapsulation efficiencies percent (EE%) were evaluated (Table 2).

#### 3.1.1. Particle Size of the Prepared LTZSPs and QuSPs

As shown in Table 2, the particle size of the prepared LTZSPs (L1-L9) ranged between 129 ± 2.6 nm and 310 ± 8.4 nm. As clearly noticed, using different types of the EA in the formulations significantly (*p* < 0.001) affected the size of the produced particles due to the difference in alkyl chain length and HLB values of EAs. Formulations L2, L5, and L8 containing Brij 35 exhibited the smallest particle size compared to those containing Tween 80 (formulations L1, L4, and L7) and Cremophor RH40 (formulations L3, L6, and L9). Brij 35 has the shortest alkyl chain length (C = 12) and the least bulky structure compared to Cremophor RH40 (bulky branched structure) [40] and Tween 80 (C = 18) [41]. In addition, the average particle size of LTZSPs was significantly decreased (*p* < 0.001) when increasing the amount of EA (Tween 80, Brij 35 and Cremophor RH40) from 20% (L1–L3) to 40% (L4–L6). Increasing the amount of EAs in the formulation caused a reduction in the interfacial tension that facilitated particle partition and formation of smaller nanovesicles [30]. Our results are in concordance with previously reported data [40]. It is noteworthy that the increase in EA concentration was on the expenses of Span 60 concentration, which is another factor that was shown previously to decrease the vesicles particle size [42]. Increasing the amount of LTZ used in the spanlastics preparation from 10 mg (L4–L6) to 15 mg (L7–L9) increased the size of the particles significantly (*p* < 0.001) due to the increased concentration of the drug loaded in the formulation that will expand the size of the vesicles [43], except in the case of Tween 80, where increasing amount of drug did not show significant differences. The particle size of the prepared QuSPs (Q1–Q8) ranged between 240 ± 80 nm and 560.3 ± 76 nm. Spanlastic formulations that were prepared using Tween 80 (Q1, Q3, Q5, and Q7) exhibited smaller particle size (statistically insignificance *p* > 0.05), compared to those prepared using Cremophor RH40 (Q2, Q4, Q6, and Q8). This could be due to the bulky branched structure of Cremophor RH40 molecules that could lead to the production of larger particle size of the spanlastic formulations [44]. In addition, the increase in the amount of Tween 80 from 20 mg (Q1 and Q5) to 40 mg (Q3 and Q7) produced spanlastics with smaller sizes (statistically insignificance *p* > 0.05), which could be attributed to the interfacial tension reduction observed with higher amounts of Tween 80, as mentioned earlier. On the contrary, increasing the amount of Cremophor RH40 from 20 mg (Q2 and Q6) to 40 mg (Q4 and Q8) increased (statistically insignificance *p* > 0.05) the size of the prepared spanlastics, which could be attributed to its bulky structure. The particle size of the prepared QuSPs was increased insignificantly (*p* > 0.05) when increasing the loaded amount of Qu from 5 mg (Q1–Q4) to 10 mg (Q5–Q8). The prepared spanlastic formulations showed PDI values ranging from 0.2 ± 0.06 to 0.7 ± 0.01, indicating that they were relatively heterogeneous [40,45].

#### 3.1.2. Encapsulation Efficiencies Percent (EE%) of the Prepared LTZSPs and QuSPs

As shown in Table 2, spanlastic formulations exhibited high EE% in the range of 94.3 ± 0.5% to 97.2 ± 0.8% for LTZSPs and 97.9 ± 0.2% to 99.6 ± 0.1% for QuSPs formulations, respectively. The type and amount of EA affected the EE% of the formulations. Formulations containing Tween 80 (L1, L4 and L7) provided an insignificantly higher (*p* > 0.05) drug encapsulation efficiency, compared to those containing Brij 35 (L2, L5, and L8) or Cremophor RH40 (L3, L6, and L9). This could be attributed to the relatively lower HLB value of Tween 80 (15), compared to the other studied EAs, in addition to its longer carbon chain length (C18) [32,46]. Changing the amount of EA showed no significant differences (*p* > 0.05) in the drug EE% of the spanlastics. Likewise, increasing the LTZ amount from 10 mg (L4 and L5) to 15 mg (L7 and L8) led to an insignificant increase (*p* > 0.05) in their EE%. In contrast, the drug EE% of formulations containing Cremophor RH40 (L9) increased significantly (*p* < 0.05) with increasing the LTZ amount.

For QuSPs formulations containing different amounts of Tween 80 and Cremophor RH40, increasing the EA concentration showed an insignificant increase in drug EE% (*p* > 0.05). Regarding the amount of Qu, the EE% increased significantly (*p* < 0.05) with increasing the amount of drug from 5 mg to 10 mg in case of the formulation containing Tween 80 (Q5 and Q7), while there was no significant difference (*p* > 0.05) between formulations containing Cremophor RH40 (Q6 and Q8).

#### 3.1.3. Zeta Potential of the Prepared LTZSPs and QuSPs

All the prepared spanlastic formulations showed negative zeta potential values (Table 2). The high charge on the vesicle surface causes repulsion between them, allowing them to be stable without agglomeration and provides a uniformly distributed suspension [30,47]. The type of EA in LTZSPs had a great influence on the zeta potential of the vesicles. Thus, zeta potential ranged from −19.9 ± 0.6 mV to −43.8 ± 0.7 mV in case of Tween 80, from −25.4 ± 1.6 mV to −39.6 ± 1.8 mV in case of Brij 35, and from −7.8 ± 1.3 mV to −21.6 ± 0.4 mV in case of Cremophor RH40. The differences in zeta potential between these three EAs were statistically significant (*p* < 0.05), due to their different HLB values. In addition, the increase in EA concentration resulted in a significant increase in zeta potential for the three studied EAs (*p* < 0.001, *p* < 0.001 and *p* < 0.005 for Tween 80, Brij 35 and Cremophor RH40, respectively). This could be attributed to the decreased size of the spanlastics when increasing the amount of EA, resulting in slower migration velocity of the charged particles and higher zeta potential value [48]. In contrast, increasing the amount of LTZ in the formulated spanlastics decreased their zeta potential values (*p* < 0.001, *p* = 0.064 and *p* = 0.001 for Tween 80, Brij 35, and Cremophor RH40, respectively). This could be attributed to the basic drug nature that might reduce the value of the negative zeta potential when increasing its amount in the formulation. Zeta potential values of QuSPs ranged from −17.6 ± 1.6 mV to −33.2 ± 2.0 mV. The formulations containing Tween 80 had significantly higher (*p* < 0.05) zeta potential values, compared to those containing Cremophor RH40. This could be attributed to the different HLB value of the used EA; the zeta potential of spanlastics increases with lower HLB value (in case of Tween 80), as more -OH ion adsorption occurs at the interface of the hydration medium [49]. The zeta potential values were significantly decreased (*p* < 0.05) with increasing the amount of EA (from 20 mg to 40 mg), which could be due to the formation of more hydrogen bonding with water molecules, due to the presence of (CH_2_-CH_2_-O)_n_ group in their structure [49]. The opposite effect was observed with increasing the amount of Qu from 5 mg to 10 mg that increased the zeta potential values, which might be due to the acidic nature of the drug.

Based on the aforementioned data, LTZSPs (L8) and QuSPs (Q5) were selected for further investigations. These formulations showed high zeta potential values that impart good stability (−35.5 for L8 and −33.23 for Q5) with homogenous size distribution (PDI = 0.4 for L8 and PDI = 0.2 for Q5), high EE% (96.3% for L8 and 99.1% for Q5), and reasonable average particle size (164.9 nm for L8 and 450.1 nm for Q5). These parameters could increase the potential of these spanlastics as transdermal drug delivery systems.

#### 3.1.4. TEM Measurements

**Figure 1:**
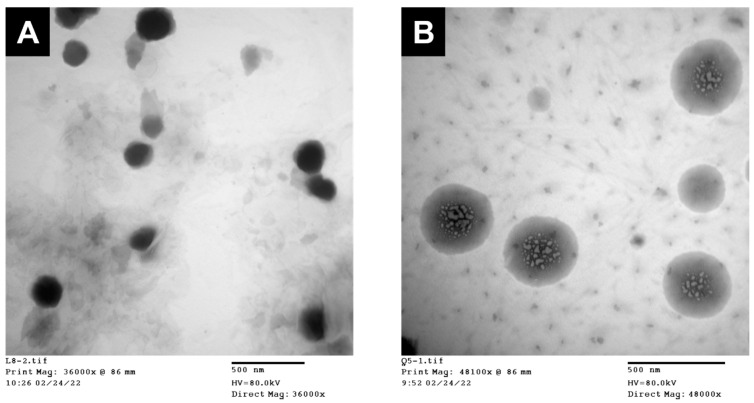
Representative transmission electron microscope images of the selected formulations (**A**) LTZSPs (formulation L8) and (**B**) QuSPs (formulation Q5).

Representative TEM photomicrographs of the selected LTZSPs (L8) and QuSPs (Q5) show homogenous, non-aggregating, and spherically shaped nanoparticles with sharp boundaries (Figure 1A and Figure 1B, respectively). The spherical shape of the drug-loaded spanlastics could be attributed to the amphiphilic nature of the non-ionic surfactants used in spanlastic preparation, which form a closed bilayer vesicles in water and reduce their surface-free energy [50]. The size of LTZSPs (~100 nm) and QuSPs (~250 nm) obtained from TEM measurements is smaller than that obtained from DLS measurements (164.9 nm and 450.1 nm, respectively), which could be attributed to the different measurement conditions [51,52]. DLS gives the average particle size of hydrated particles, while TEM measures the size of dried ones, resulting in a smaller particle size for the latter.

#### 3.1.5. Fourier-Transform Infrared (FTIR) Spectroscopy Studies

**Figure 2:**
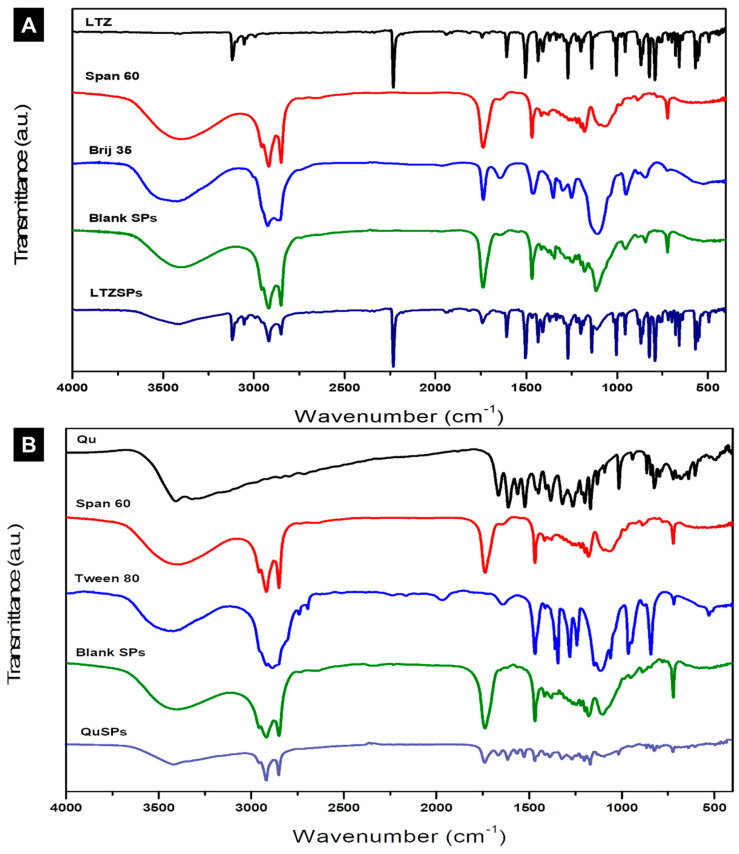
FTIR spectra of (**A**) letrozole (LTZ), Span 60, Brij 35, blank spanlastics (Blank SPs), and letrozole loaded spanlastics (LTZSPs, formulation L8). (**B**) Quercetin (Qu), Span 60, Tween 80, blank spanlastics (Blank SPs), and quercetin-loaded spanlastics (QuSPs, formulation Q5).

The selected LTZSPs and QuSPs formulations and their individual components were analyzed using FTIR spectroscopy and the spectra are shown in Figure 2A and Figure 2B, respectively. The FTIR spectrum of LTZ displays its characteristic absorption bands at 2229 cm^−1^ for C≡N stretching, 1263 cm^−1^ for C–N stretching, and 690–900 cm^−1^ for out of plane C–H bending [53]. The FTIR spectrum of Qu shows stretching of the -OH groups at 3406 and 3283 cm^−1^, bending of phenolic -OH at 1379 cm^−1^, C=O aryl ketonic stretching absorption at 1666 cm^−1^, and C=C aromatic ring stretching bands at 1666, 1610, 1560, and 1510 cm^−1^. Moreover, the spectrum displays absorption bands due to in-plane bending at 1317 cm^−1^ and out-of-plane bending at 933, 820, 679, and 600 cm^−1^ of the C–H in the aromatic hydrocarbon. The C–O stretching in the aryl ether ring, the C–O stretching in phenol, and the C–CO–C stretch and bending in ketone display bands at 1263, 1200, and 1165 cm^−1^, respectively [54].

The spectrum of Span 60 shows characteristic bands, such as aliphatic O-H stretching at 3410 cm^−1^, asymmetric and symmetric aliphatic C–H stretching at 2916 and 2849 cm^−1^, respectively, and C=O stretching of the ester at 1745 cm^−1^ [55]. Tween 80 spectrum shows bands at 2907 and 2855 cm^−1^ related to the asymmetric and symmetric vibration of methylene (-CH_2_), respectively. The band at 1735 cm^−1^ is caused by the ester group’s C=O stretching. The O-H stretching vibration is responsible for the strong band around 3436 cm^−1^ [30]. The FTIR spectrum of Brij 35 shows its characteristic bands at 2882 cm^−1^, 1965 cm^−1^, and 1101 cm^−1^ for -C=CH_3_ stretching, carbonyl band, and C-O stretching, respectively [56]. The FTIR spectrum of blank spanlastic formulations shows the characteristic bands of both Span 60 and Brij 35 in the case of LTZSPs and the characteristic bands of Span 60 and Tween 80 in case of QuSPs. However, the bands show lower intensity compared with the same bands of the individual components. The lipid bilayer formulation is responsible for the lower intensity of the bands in the blank spanlastic formulations [57]. Furthermore, the characteristic bands of either LTZ or Qu and different formulation excipients that were used in the optimized spanlastic formulations are shown in the FTIR spectrum, indicating that there were no interactions between the drug and the different excipients. The observed slight shifting and decreased intensity of the bands may be attributed to hydrogen bond formation, Van der Wall forces, or dipole interactions between either LTZ or Qu and other excipients, improving both drug encapsulation and nano-vesicle stability [58,59].

### 3.2. In Vitro Drug Release Study

**Figure 3:**
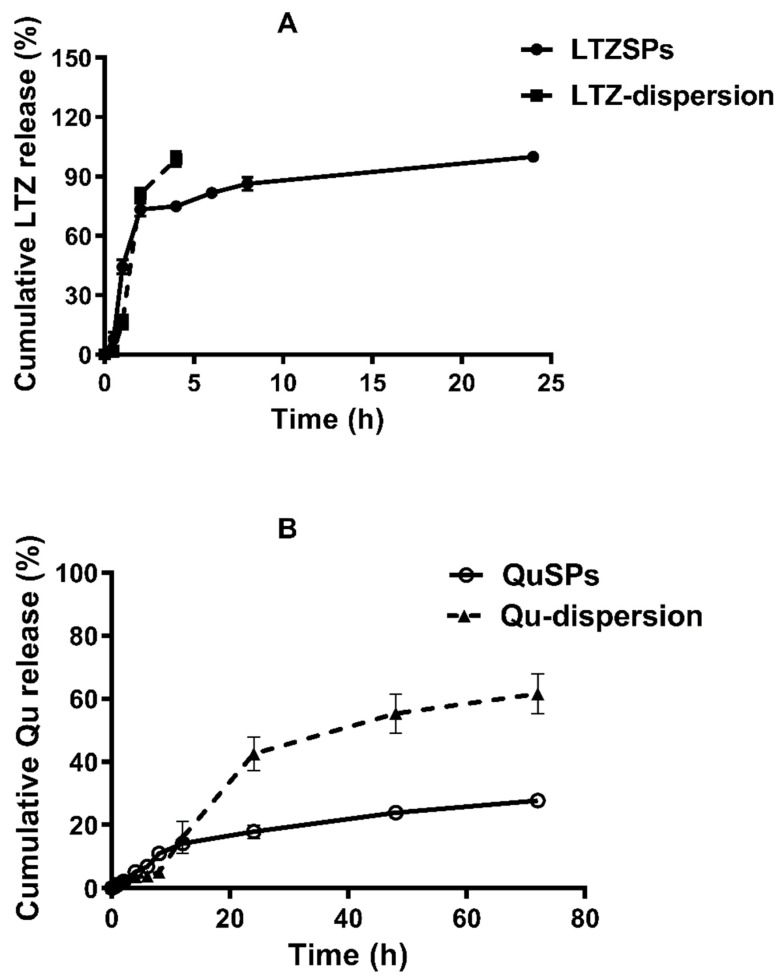
Cumulative in vitro release profiles of (**A**) The optimized LTZSPs compared to free LTZ dispersion in hydroalcoholic PBS solution pH 7.4 at 37 °C. (**B**) The optimized QuSPs compared to free Qu dispersion in PBS pH 7.4 plus 1% Tween 80 at 37 °C. Data are expressed as mean ± SD (*n* = 3). Abbreviations: LTZ; letrozole, Qu; quercetin, LTZSPs; letrozole-loaded spanlastics, QuSPs; quercetin-loaded spanlastics.

The in vitro release profiles of LTZSPs and QuSPs are depicted in Figure 3A and Figure 3B, respectively. The individual dispersions of free LTZ or free Qu were used as controls to confirm the release of the drug through the dialysis membrane and to investigate the effect of drug encapsulation into spanlastics on its release profiles. LTZ aqueous suspension exhibited cumulative percent drug release of 98.8 ± 3.7% (0.74 ± 0.03 mg) after 4 h. LTZ release was fast from the aqueous suspension as it was only governed by the dissolution rate of the drug. In contrast, LTZ loaded into spanlastic preparations showed a more controlled drug release (cumulative drug release of 75.0 ± 1.4% (0.56 ± 0.01 mg) after 4 h). At the end of 24 h, the corresponding percent drug released was 99.9 ± 1.2% (0.75 ± 0.01 mg). The release of drug from spanlastic formulations was influenced by the attractive forces within the phospholipids bilayer that resulted in a more delayed drug release [51].

Qu dispersion showed a maximum drug release of 61.5 ± 6.3% (0.62 ± 0.06 mg) after 72 h. Regarding Qu release from spanlastics, a slow drug release pattern was observed with an initial percent drug release of 10.9 ± 1.1% (0.11 ± 0.02 mg) after 8 h, followed by a more controlled sustained release of 27.7 ± 1.2% (0.28 ± 0.01 mg) after 72 h. The entire amount of loaded drug was not released from the vesicles. This might be due to entrapment of the drug in the lipophilic region of the vesicles [60]. The slower release of both LTZ and Qu from spanlastics compared with the free drugs could be attributed to the high transition temperature and the long-chain length of the non-ionic surfactant Span 60, which leads to formation of a more rigid, less permeable bilayer [34]. These results were consistent with previous studies, which indicated that spanlastics had sustained release profiles compared to free drug dispersion [36,61].

### 3.3. Ex Vivo Permeation Study

**Figure 4:**
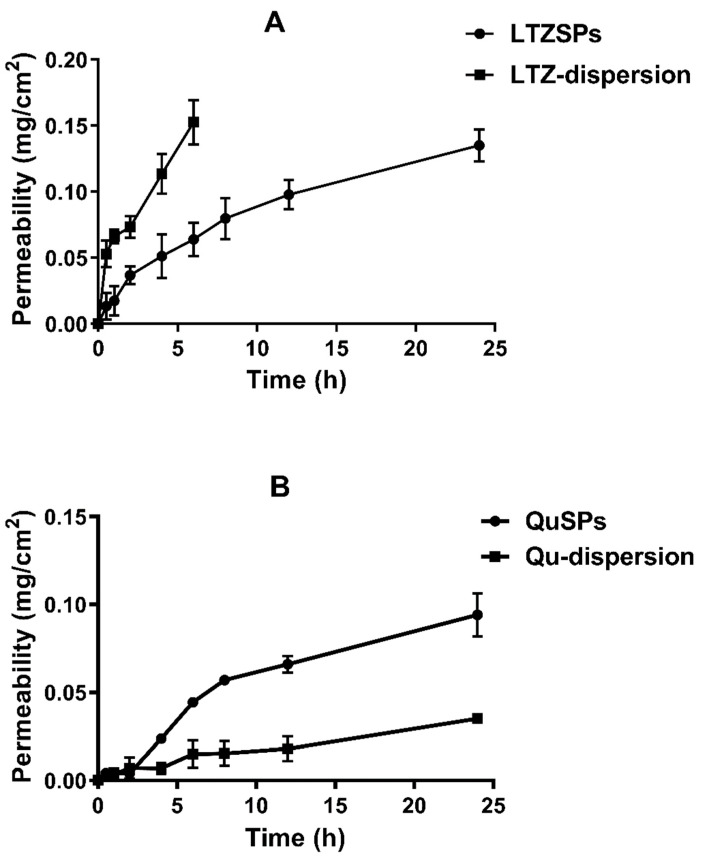
Ex vivo permeation profile of (**A**) The optimized LTZSPs (formulation L8), compared to free LTZ dispersion in hydroalcoholic PBS solution pH 7.4 at 37 °C. (**B**) The optimized QuSPs (formulation Q5), compared to free Qu dispersion in PBS pH 7.4 plus 1% Tween 80 at 37 °C. Data are presented as mean ± SD, *n* = 3. Abbreviations: LTZ; letrozole, Qu; quercetin, LTZSPs; letrozole-loaded spanlastics, QuSPs; quercetin-loaded spanlastics.

Freshly excised rat skin was used as an in vitro model for comparing transdermal permeation properties of LTZSPs and QuSPs versus free LTZ and Qu dispersions to give an insight into the skin permeability properties of the optimized spanlastic formulations. In comparison to the free drug dispersion, the quantitative mass transfer of LTZ across skin was lower when administered in form of LTZSPs (Figure 4A and Table 3). The LTZ cumulative percent permeated as free drug dispersion was 99.8 ± 1.5% (0.153 ± 0.017 mg/cm^2^) after 6 h, compared to 41.8 ± 7.9% (0.064 ± 0.013 mg/cm^2^) for the LTZSPs formulation at the same time. The maximum percent of drug permeated after 24 h from optimized LTZSPs was 88.4 ± 10.9% (0.135 ± 0.012 mg/cm^2^). LTZSPs exhibited a sustained permeation of the drug over the LTZ dispersion. This could be explained by the entrapment of LTZ within the vesicular structure of spanlastics leading to slow flux and permeability for 24 h. On the contrary, free LTZ from the LTZ dispersion could permeate freely through the skin [62,63].

Qu permeability (mg/cm^2^) from spanlastics formulation Q5 in comparison with drug dispersion is shown in Figure 4B. The optimized QuSPs had a significant increase (*p* < 0.01) in cumulative percent drug permeated when compared to free drug dispersion with 27.9 ± 0.6% (0.057 ± 0.001 mg/cm^2^) drug permeation after 8 h, compared to only 7.5 ± 0.9% (0.015 ± 0.007 mg/cm^2^) from the free drug dispersion after the same time. The maximum percent of drug permeated after 24 h from the optimized QuSPs was approximately 2.7-fold higher than that from free Qu dispersion. The apparent permeability coefficient (*P_app_*) value for QuSPs was about 3.8-fold higher than that from the free drug suspension. The in vitro permeation parameters also revealed that QuSPs showed significantly higher (*p* < 0.05) transdermal flux (*J_ss_*) compared to Qu dispersion (Table 3). The enhanced Qu permeability from the vesicles could be attributed to multiple factors, including the nanosized vesicle diameter and the presence of surfactants. Nonionic surfactants within SPs formulation could impart elasticity and deformability to the vesicles, which enhances their ability to squeeze themselves through the intercellular space and augment drug permeability [30,38,61,64]. As shown in Supplementary Materials Figure S1, high proportions of LTZ/Qu were permeated from LTZSPs/QuSPs through rat skin after 24 h incubation (88.4 ± 11% and 46.2 ± 0.11%, respectively), which could be sufficient for their therapeutic effect.

It is noteworthy that the skin permeability profiles were different for LTZSPs and QuSPs where the former had much slower drug permeation. The reason behind this is not clear but might be related to the different physicochemical properties of the two drugs. Based on the aforementioned experimental findings, the combined application of LTZSPs and QuSPs as transdermal delivery systems could have significant potential in alleviating breast cancer development and progression. 

### 3.4. Cell Viability Assay of LTZSPs, QuSPs, and Their Combinatorial Treatment

**Figure 5:**
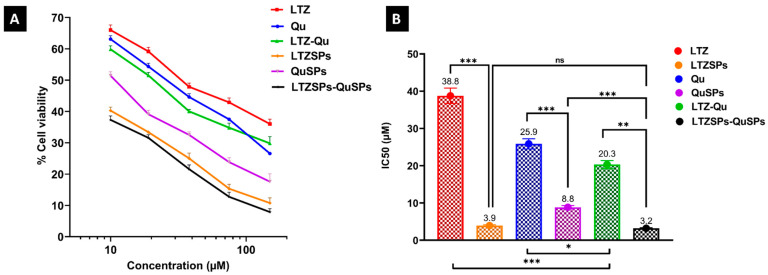
Cytotoxicity assessment using MTT assay against MCF-7 cell line. (**A**) Dose–response curves of MCF-7 cells to treatment with LTZ, Qu, and their combination and also LTZSPs, QuSPs, and their combination using indicated concentration range using an MTT assay after 48 h of treatment. (**B**) The mean IC_50_ of LTZ, Qu, and their combination and also LTZ-Qu, LTZSPs, and their combination against MCF-7 cells. Data are expressed as mean ± SD (*n* = 3). *** *p* < 0.001, ** p < 0.01, * *p* < 0.05. Abbreviations: LTZ; letrozole, Qu; quercetin, LTZSPs; letrozole-loaded spanlastics, QuSPs; quercetin-loaded spanlastics, ns; not significant.

The cytotoxic activity of the selected LTZSPs and QuSPs against MCF-7 breast cancer cells was investigated in vitro to evaluate the impact of the nanospanlastic delivery systems on enhancing the cytotoxicity of soluble LTZ and Qu using MTT assay. The results showed enhanced cytotoxic activity and marked a statistically significant drop in the IC_50_ after incorporation into spanlastics, compared to the free soluble LTZ and Qu (Figure 5A,B). The IC_50_ values of the soluble LTZ, Qu, and their combination were decreased significantly (*p* < 0.001) after being formulated; from 38.8 ± 2.0, 25.9 ± 1.4, and 20.3 ± 1.0 µM to 3.9 ± 0.2, 8.8 ± 0.5, and 3.2 ± 0.2 µM, respectively. The enhanced cytotoxic effect of the drugs encapsulated into spanlastics could be due to either the added cytotoxic effect of blank (drug-free) spanlastics or the drug-loaded spanlastics. To exclude the effect of blank spanlastics, we measured their IC_50_ and found it to be 192 ± 10 µM, 108 ± 6 µM, and 215 ± 11 µM for letrozole-free spanlastics, quercetin-free spanlastics and their combination, respectively. This is compared to 3.9 ± 0.2 µM for LTZSPs, 8.8 ± 0.5 µM for QuSPs, and 3.2 ± 0.2 µM for their combination. Looking into this difference in IC_50_, it could be concluded that the enhanced IC_50_ of the drug-loaded spanlastics is due to nanoparticle cellular uptake. This assumption is supported by other reported findings where the better cytotoxic performance of the drug-loaded spanlastics was attributed to the protective role of the nanocarrier with their higher permeability through the cell membrane due to its elasticity and the presence of edge activators [51,64,65]. Previous studies have reported that Span-60 containing nano-formulations showed enhanced cellular uptake with improved cytotoxic activity against MCF-7 breast cancer cell line [66,67]. Mehanna, et al. [68] also reported that the enhanced anticancer effect of thymoquinone-loaded nanoparticles is due to the high capacity of nanoparticle to be internalized and localized inside the cellular target of MCF-7 breast cancer cells.

Combination strategy is important to overcome the predisposed resistance to the aromatase inhibitor, LTZ. The cytotoxic activity of the combination treatment of the soluble LTZ and Qu or LTZSPs and QuSPs against MCF-7 breast cancer cells was investigated and compared to the single treatment of each. A more pronounced cytotoxic activity was achieved by the combinatorial treatment of soluble Qu and LTZ or QuSPs and LTZSPs at the studied concentration range (Figure 5A). Additionally, a significant 2-fold (*p* < 0.001) and 1.3-fold (*p* < 0.05) decrease in the IC_50_ of soluble LTZ and Qu combination was obtained compared to the IC_50_ of their corresponding individual drugs; LTZ and Qu, respectively (Figure 5B). Furthermore, the LTZSPs and QuSPs combination was assessed using CompuSyn software for their synergism, the resulting combination index (CI) showed a synergistic pattern (CI < 1) at high drug doses (with high effect level) (Figure S2), which is more relevant to the anticancer therapy than the small drug doses [1].

### 3.5. Cell Cycle Analysis by Flow Cytometry

**Figure 6:**
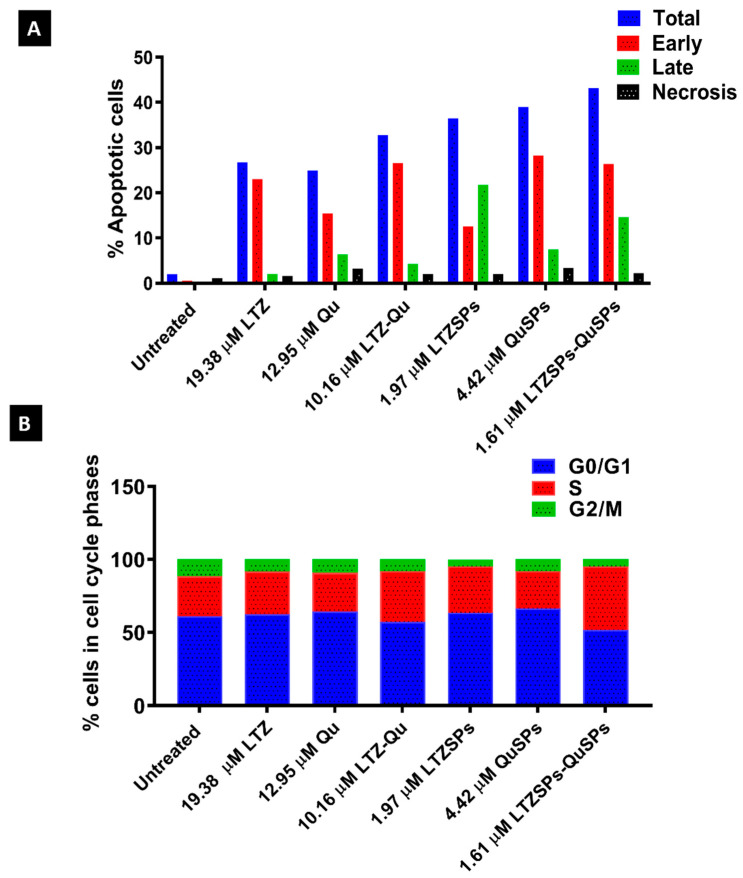
Cell cycle studies of MCF-7 cells treated with soluble LTZ, Qu, and their combination and also LTZSPs, QuSPs, and their combination using indicated concentrations. (**A**) Percentage of apoptotic cells. (**B**) Representative histograms indicating percentage of MCF-7 cells in each cell cycle. Abbreviations: LTZ; letrozole, Qu; quercetin, LTZSPs; letrozole-loaded spanlastics, QuSPs; quercetin-loaded spanlastics.

Flow cytometry and annexin V/PI staining were applied to determine whether the cytotoxic effect of LTZ, Qu or their combination and LTZSPs, QuSPs or their combination on MCF-7 cell growth is due to the induction of apoptosis or not. The concentrations of the treatment in this study were selected according to our previous cytotoxicity results and below their obtained individual IC_50_. The treatment of MCF-7 cells with LTZ, Qu, or their combination resulted in an enhanced apoptosis and necrosis compared to untreated cells (Figure 6A). The percentage of necrotic and apoptotic cell population was increased from 1.96% for the control MCF-7 cells to 32.81% for soluble LTZ and Qu combination. Moreover, the use of this combination in the form of spanlastics increased the percentage of apoptotic and necrotic cells to 43.18%. As shown in Figure 6B and Figure S3, Qu and QuSPs arrested MCF-7 cells at G_0_/G_1_ phase (64.28% and 66.41%, respectively) compared to control cells, while LTZ and LTZSPs arrested the cell growth at G_0_/G_1_ phase (62.47% and 63.57%, respectively) and S phase (29.26% and 31.72%, respectively). However, the combination of LTZSPs and QuSPs had a more pronounced increase in the S phase apoptosis (43.55%), compared with that of the soluble Qu and LTZ combination (34.81%), indicating higher growth arrest at this stage.

Qu is a flavonoid antioxidant that promotes apoptosis, which is the ultimate objective in cancer treatment [69], and previous research reported its effect on the reduction in MCF-7 cells viability [70]. Our results showed enhanced percentage of necrosis in MCF-7 cells after treatment using Qu either alone or combined with LTZ, which is similar to the previously reported results [71]. Flow cytometry results showed that Qu arrested the MCF-7 cell growth at G_1_ check point while LTZ arrested cell growth at G_1_ and S phase, which is consistent with previous reports [25,72].

Previously, the combination of Qu and LTZ with lower doses was highly effective on cell apoptosis through the induction of mitochondrial apoptosis [15]. Our results showed that the effect of Qu and LTZ combinatorial treatment with lower concentrations induced higher necrosis and apoptosis than each single drug. Moreover, an increase in the MCF-7 cell necrosis and apoptosis was obtained after cell treatment using the drug-loaded spanlastics, compared to their free counterpart. The observed enhanced cell cytotoxicity, cell necrosis, and apoptosis are probably attributed to the improved drug permeability and cell membrane diffusion that enhanced the drug accumulation inside the cells. This is due to the presence of edge activators in spanlastic formulations, which enhances the elasticity and deformability of vesicles [51].

### 3.6. Assessment of Reactive Oxygen Species (ROS)

**Figure 7:**
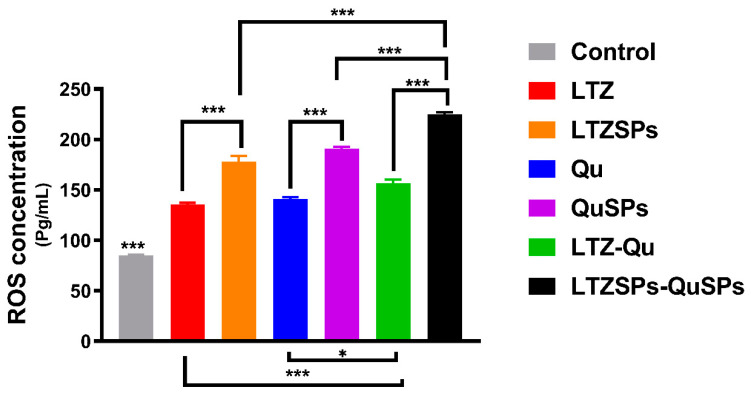
ROS levels using ELISA assay against MCF-7 cell line. MCF-7 cells were treated with selected concentrations of LTZ and Qu and their combination and also LTZ–Qu, LTZSPs, QuSPs and their combination and measured using an ELISA after 24 h of treatment. All samples show significance difference compared to control. Data are expressed as mean ± SD (*n* = 3). *** *p* < 0.001, * *p* < 0.05. Abbreviations: LTZ; letrozole, Qu; quercetin, LTZSPs; letrozole-loaded spanlastics, QuSPs; quercetin-loaded spanlastics.

ROS act as vital intracellular secondary messengers for numerous cytokines and growth factors in cancer cells, thereby they have a strong pro-survival effect when present in controlled levels. However, elevated ROS levels can trigger cellular damage and even cell death [73]. ROS levels using ELISA assay against MCF-7 cell line are shown in Figure 7. Both LTZ and Qu either free or loaded in spanlastics significantly (*p* < 0.001) increased ROS levels in the MCF-7 cells compared to control. The combination of LTZSPs and QuSPs showed 1.4-fold enhanced ROS levels compared with the soluble LTZ–Qu combination.

The enhanced ROS levels in MCF-7 cancer cells produced by either LTZ or LTZSPs could be attributed to the decrease in estrogen levels in the cells, which is believed to have an antioxidant effect [74,75]. Additionally, the treatment of cancer cells with Qu produces quercetin–semiquinones and quercetin–quinones, that have pro-oxidant properties inside the cells that are responsible for their anticancer activity [76]. These compounds are extremely reactive to thiols and react with reduced glutathione (GSH), causing its depletion. Cell death by apoptosis results from the disturbance of GSH antioxidant defense in cells with persistent ROS overload, such as malignant cells [73]. The combination of LTZ and Qu offers two distinct mechanisms to generate high ROS levels inside cancer cells, which might reduce the needed dose, reduce the adverse effects, and improve the patient compliance.

## 4. Conclusions

LTZ and Qu were successfully loaded into spanlastics having high drug encapsulation efficiency, relatively small size, and negative zeta potential. The results of the in vitro cell cytotoxicity study highlight the potential of combinatorial treatment of LTZ and Qu in the improvement of breast cancer treatment outcomes. Moreover, loading these drugs into spanlastics substantially enhanced the drugs’ cytotoxic effects at much lower doses, compared to their soluble free drug counterparts. Therefore, the transdermal delivery of LTZSPs and QuSPs could be a promising site-specific delivery approach to enhance their cytotoxic effects and reduce the harmful adverse effects.

## Figures and Tables

**Table 1:** Compositions of the prepared LTZSPs and QuSPs.

No.	Formulation Code	Drug (mg/mL)		EA	EA (mg/mL)	Span 60 (mg/mL)	EA/Span 60 Ratio
		LTZ	Qu				
1	L1	1.0		Tween 80	2.0	8.0	20/80
2	L2	1.0		Brij 35	2.0	8.0	20/80
3	L3	1.0		Cremophor RH40	2.0	8.0	20/80
4	L4	1.0		Tween 80	4.0	6.0	40/60
5	L5	1.0		Brij 35	4.0	6.0	40/60
6	L6	1.0		Cremophor RH40	4.0	6.0	40/60
7	L7	1.5		Tween 80	4.0	6.0	40/60
8	L8	1.5		Brij 35	4.0	6.0	40/60
9	L9	1.5		Cremophor RH40	4.0	6.0	40/60
10	Q1		0.5	Tween 80	2.0	8.0	20/80
11	Q2		0.5	Cremophor RH40	2.0	8.0	20/80
12	Q3		0.5	Tween 80	4.0	6.0	40/60
13	Q4		0.5	Cremophor RH40	4.0	6.0	40/60
14	Q5		1.0	Tween 80	2.0	8.0	20/80
15	Q6		1.0	Cremophor RH40	2.0	8.0	20/80
16	Q7		1.0	Tween 80	4.0	6.0	40/60
17	Q8		1.0	Cremophor RH40	4.0	6.0	40/60

The total amount of LTZ in the prepared LTZSPs is 10 mg (1 mg/mL) and 15 mg (1.5 mg/mL) while the total amount of Qu in the prepared QuSPs is 5 mg (0.5 mg/mL) and 10 mg (1 mg/mL). Abbreviations: LTZSPs; letrozole-loaded spanlastics, QuSPs; quercetin-loaded spanlastics, EA; edge activator.

**Table 2:** Characterization of the prepared LTZSPs and QuSPs.

Formulations	* Average Particle Size (nm) ± SD	PDI ± SD	Zeta Potential (mV) ± SD	EE% ± SD
L1	242.1 ± 8.2	0.5 ± 0.06	−19.9 ± 0.6	95.8 ± 0.5
L2	240.3 ± 10.1	0.5 ± 0.03	−25.4 ± 1.6	94.3 ± 0.5
L3	310.3 ± 8.4	0.4 ± 0.02	−9.9 ± 1.7	95.4 ± 0.8
L4	192.0 ± 3.5	0.4 ± 0.04	−43.8 ± 0.7	96.8 ± 1.2
L5	129.0 ± 2.6	0.4 ± 0.01	−39.6 ± 1.8	95.3 ± 0.5
L6	257.0 ± 1.7	0.4 ± 0.02	−21.6 ± 0.4	94.5 ± 0.6
L7	188.5 ± 3.7	0.4 ± 0.01	−28.8 ± 0.6	97.2 ± 0.8
L8	164.9 ± 4.8	0.4 ± 0.01	−35.5 ± 2.1	96.3 ± 0.3
L9	289.7 ± 3.9	0.4 ± 0.04	−7.8 ± 1.3	97.1 ± 0.5
Q1	367.2 ± 40.1	0.5 ± 0.06	−26.5 ± 1.4	99.4 ± 0.1
Q2	406.4 ± 51.7	0.6 ± 0.08	−19.2 ± 0.5	99.6 ± 0.1
Q3	240.0 ± 79.6	0.6 ± 0.11	−22.5 ± 0.6	97.9 ± 0.2
Q4	560.3 ± 76.0	0.5 ± 0.06	−17.6 ± 1.6	99.5 ± 0.2
Q5	450.1 ± 45.4	0.2 ± 0.06	−33.23 ± 2.0	99.1 ± 0.1
Q6	480.6 ± 3.9	0.4 ± 0.04	−23.6 ± 1.4	99.5 ± 0.1
Q7	434.9 ± 91.3	0.6 ± 0.05	−23.2 ± 0.2	99.0 ± 0.2
Q8	498.4 ± 30.0	0.7 ± 0.01	−18.6 ± 1.6	99.4 ± 0.2

* Average particle size as measured by dynamic light scattering. Abbreviations: LTZSPs; letrozole loaded spanlastics, QuSPs; quercetin loaded spanlastics, PDI; polydispersity index, EE%; Encapsulation efficiency percentage. All data are presented as the mean ± SD (*n* = 3).

**Table 3:** Permeation parameters of LTZSPs and QuSPs versus LTZ and Qu dispersions across rat skin.

Formulation	*P_app_* (×10^−6^) ± SD (×10^−6^) cm/s	*J_ss_* (mg cm^−2^ h^−1^)
LTZSPs	2.25 ± 1.08	0.006 ± 0.003
LTZ dispersion	7.95 ± 1.67 **	0.021 ± 0.005 **
QuSPs	1.45 ± 0.28 **	0.005 ± 0.001 **
Qu dispersion	0.39 ± 0.02	0.0014 ± 0.00007

Each value represents mean ± SD (*n* = 3). Abbreviations: *P_app_*, apparent permeability coefficient; *J_ss_*, steady state flux. ** LTZ dispersion significantly different compared with LTZSPs and QuSPs significantly different compared with Qu dispersion (*p* < 0.01).

## Data Availability

Not applicable.

## Supplementary Materials

The following supporting information can be downloaded at https://www.mdpi.com/article/10.3390/pharmaceutics14081727/s1. Figure S1: Proportions of the permeated amount of (A) LTZ from LTZSPs and LTZ-dispersion (B) Qu from QuSPs and Qu-dispersion through rat skin after 24-h incubation. Skin: stratum corneum, epidermis and dermis, while transdermal: receptor chamber. Abbreviations: LTZ; letrozole, Qu; Quercetin, LTZSPs; letrozole loaded spanlastics, QuSPs; Quercetin loaded spanlastics. Figure S2: Combination index versus fraction affected plot for LTZSPs and QuSPs combination. Abbreviations: Fa; fraction affected, LTZSPs; letrozole-loaded spanlastics, QuSPs; quercetin-loaded spanlastics. Figure S3: Cell cycle profile of MCF-7 cells treated with soluble LTZ, Qu, and their combination, LTZSPs, QuSPs and their combination.

## References

[1] Houghton S.C., Hankinson S.E. (2021). Cancer progress and priorities: Breast cancer. Cancer Epidemiol. Biomark. Prev..

[2] Society A.C. (2019). Breast Cancer Facts & Figures 2019–2020. https://www.cancer.org/research/cancer-facts-statistics/breast-cancer-facts-figures.html.

[3] Ahmad A. (2019). Breast Cancer Metastasis and Drug Resistance: Challenges and Progress.

[4] Hart C.D., Migliaccio I., Malorni L., Guarducci C., Biganzoli L., Di Leo A. (2015). Challenges in the management of advanced, ER-positive, HER2-negative breast cancer. Nat. Rev. Clin. Oncol..

[5] Li L., Xu X., Fang L., Liu Y., Sun Y., Wang M., Zhao N., He Z. (2010). The transdermal patches for site-specific delivery of letrozole: A new option for breast cancer therapy. AAPS PharmSciTech.

[6] Fabian C.J. (2007). The what, why and how of aromatase inhibitors: Hormonal agents for treatment and prevention of breast cancer. Int. J. Clin. Pract..

[7] Maniyar M., Chakraborty A., Kokare C. (2020). Formulation and evaluation of letrozole-loaded spray dried liposomes with PEs for topical application. J. Liposome Res..

[8] Cavadias I., Rouzier R., Lerebours F., Héquet D. (2020). Hot flushes and breast cancer with positive hormone receptors: Mechanisms and management. Bull. Cancer.

[9] Nunes F.A.P., de Farias M.L.F., Oliveira F.P., Vieira L.N., Lima L.F.C., de Paula Paranhos F.N., de Mendonça L.M.C., Madeira M. (2021). Use of aromatase inhibitors in patients with breast cancer is associated with deterioration of bone microarchitecture and density. Arch. Endocrinol. Metab..

[10] Xi H., Yang Y., Zhao D., Fang L., Sun L., Mu L., Liu J., Zhao N., Zhao Y., Zheng N. (2010). Transdermal patches for site-specific delivery of anastrozole: In vitro and local tissue disposition evaluation. Int. J. Pharm..

[11] Regenthal R., Voskanian M., Baumann F., Teichert J., Brätter C., Aigner A., Abraham G. (2018). Pharmacokinetic evaluation of a transdermal anastrozole-in-adhesive formulation. Drug Des. Devel. Ther..

[12] Elzoghby A.O., Mostafa S.K., Helmy M.W., El Demellawy M.A., Sheweita S.A. (2017). Multi-Reservoir phospholipid shell encapsulating protamine nanocapsules for co-delivery of letrozole and celecoxib in breast cancer therapy. Pharm. Res..

[13] Jelovac D., Macedo L., Goloubeva O.G., Handratta V., Brodie A.M. (2005). Additive antitumor effect of aromatase inhibitor letrozole and antiestrogen fulvestrant in a postmenopausal breast cancer model. Cancer Res..

[14] Bonelli M.A., Fumarola C., Alfieri R.R., La Monica S., Cavazzoni A., Galetti M., Gatti R., Belletti S., Harris A.L., Fox S.B. (2010). Synergistic activity of letrozole and sorafenib on breast cancer cells. Breast Cancer Res. Treat..

[15] Yeter Ç., Özge Ç. (2021). Anticancer effect of the letrozole-quercetin combination mediated by FOXOs and estrogen receptors in breast cancer cells. J. Res. Pharm..

[16] Lamb H.M., Adkins J.C. (1998). Letrozole. Drugs.

[17] Wang R., Yang L., Li S., Ye D., Yang L., Liu Q., Zhao Z., Cai Q., Tan J., Li X. (2018). Quercetin inhibits breast cancer stem cells via downregulation of aldehyde dehydrogenase 1A1 (ALDH1A1), chemokine receptor type 4 (CXCR4), mucin 1 (MUC1), and epithelial cell adhesion molecule (EpCAM). Med. Sci. Monit. Int. Med. J. Exp. Clin. Res..

[18] Dong Y., Yang J., Yang L., Li P. (2020). Quercetin inhibits the proliferation and metastasis of human non-small cell lung cancer cell line: The key role of Src-mediated fibroblast growth factor-inducible 14 (Fn14)/nuclear factor kappa B (NF-κB) pathway. Med. Sci. Monit. Int. Med. J. Exp. Clin. Res..

[19] Ong C.S., Tran E., Nguyen T.T., Ong C.K., Lee S.K., Lee J.J., Ng C.P., Leong C., Huynh H. (2004). Quercetin-induced growth inhibition and cell death in nasopharyngeal carcinoma cells are associated with increase in Bad and hypophosphorylated retinoblastoma expressions. Oncol. Rep..

[20] Han C., Gao H., Zhang X. (2016). Quercetin anti-cancer effect in renal cancer through regulating survivin expression and caspase 3 activity. Med. One.

[21] Zhang X.-A., Zhang S., Yin Q., Zhang J. (2015). Quercetin induces human colon cancer cells apoptosis by inhibiting the nuclear factor-kappa B Pathway. Pharmacogn. Mag..

[22] Ward A.B., Mir H., Kapur N., Gales D.N., Carriere P.P., Singh S. (2018). Quercetin inhibits prostate cancer by attenuating cell survival and inhibiting anti-apoptotic pathways. World J. Surg. Oncol..

[23] Angst E., Park J.L., Moro A., Lu Q.-Y., Lu X., Li G., King J., Chen M., Reber H.A., Go V.L.W. (2013). The flavonoid quercetin inhibits pancreatic cancer growth in vitro and in vivo. Pancreas.

[24] Shafabakhsh R., Asemi Z. (2019). Quercetin: A natural compound for ovarian cancer treatment. J. Ovarian Res..

[25] Ranganathan S., Halagowder D., Sivasithambaram N.D. (2015). Quercetin suppresses twist to induce apoptosis in MCF-7 breast cancer cells. PLoS ONE.

[26] Schmitt-Ney M., Camussi G. (2015). The PAX3-FOXO1 fusion protein present in rhabdomyosarcoma interferes with normal FOXO activity and the TGF-β pathway. PLoS ONE.

[27] De Jong W.H., Borm P.J. (2008). Drug delivery and nanoparticles: Applications and hazards. Int. J. Nanomed..

[28] Tomoda K., Makino K. (2014). Nanoparticles for transdermal drug delivery system (TDDS). Colloid and Interface Science in Pharmaceutical Research and Development.

[29] Ansari M.D., Saifi Z., Pandit J., Khan I., Solanki P., Sultana Y., Aqil M. (2022). Spanlastics a novel nanovesicular carrier: Its potential application and emerging trends in therapeutic delivery. AAPS PharmSciTech.

[30] Badria F., Mazyed E. (2020). Formulation of nanospanlastics as a promising approach for improving the topical delivery of a natural leukotriene inhibitor (3- Acetyl-11-Keto-β-Boswellic Acid): Statistical optimization, in vitro characterization, and ex vivo permeation study. Drug Des. Dev. Ther..

[31] Sharma A., Pahwa S., Bhati S., Kudeshia P. (2020). Spanlastics: A modern approach for nanovesicular drug delivery system. Int. J. Pharm. Sci. Res..

[32] Mazyed E.A., Helal D.A., Elkhoudary M.M., Abd Elhameed A.G., Yasser M. (2021). Formulation and optimization of nanospanlastics for improving the bioavailability of green tea epigallocatechin gallate. Pharmaceuticals.

[33] Mohamed H.B., Attia Shafie M.A., Mekkawy A.I. (2022). Chitosan nanoparticles for meloxicam ocular delivery: Development, in vitro characterization, and in vivo evaluation in a rabbit eye model. Pharmaceutics.

[34] Abdelbari M.A., El-Mancy S.S., Elshafeey A.H., Abdelbary A.A. (2021). Implementing spanlastics for improving the ocular delivery of clotrimazole: In vitro characterization, ex vivo permeability, microbiological assessment and in vivo safety study. Int. J. Nanomed..

[35] Allam A., Elsabahy M., El Badry M., Eleraky N.E. (2021). Betaxolol-loaded niosomes integrated within pH-sensitive in situ forming gel for management of glaucoma. Int. J. Pharm..

[36] Fahmy A.M., El-Setouhy D.A., Ibrahim A.B., Habib B.A., Tayel S.A., Bayoumi N.A. (2018). Penetration enhancer-containing spanlastics (PECSs) for transdermal delivery of haloperidol: In vitro characterization, ex vivo permeation and in vivo biodistribution studies. Drug Deliv..

[37] El Maghraby G.M., Ahmed A.A., Osman M.A. (2015). Penetration enhancers in proniosomes as a new strategy for enhanced transdermal drug delivery. Saudi Pharm. J..

[38] Farghaly D.A., Aboelwafa A.A., Hamza M.Y., Mohamed M.I. (2017). Topical delivery of fenoprofen calcium via elastic nano-vesicular spanlastics: Optimization using experimental design and in vivo evaluation. AAPS PharmSciTech.

[39] Liu D., Hu H., Lin Z., Chen D., Zhu Y., Hou S., Shi X. (2013). Quercetin deformable liposome: Preparation and efficacy against ultraviolet B induced skin damages in vitro and in vivo. J. Photochem. Photobiol. B.

[40] Al-Mahallawi A.M., Khowessah O.M., Shoukri R.A. (2017). Enhanced non invasive trans-tympanic delivery of ciprofloxacin through encapsulation into nano-spanlastic vesicles: Fabrication, in-vitro characterization, and comparative ex-vivo permeation studies. Int. J. Pharm..

[41] Wang X., Gao Y. (2018). Effects of length and unsaturation of the alkyl chain on the hydrophobic binding of curcumin with Tween micelles. Food Chem..

[42] Elhabak M., Ibrahim S., Abouelatta S.M. (2021). Topical delivery of l-ascorbic acid spanlastics for stability enhancement and treatment of UVB induced damaged skin. Drug Deliv..

[43] Kheradmandnia S., Vasheghani-Farahani E., Nosrati M., Atyabi F. (2010). The effect of process variables on the properties of ketoprofen loaded solid lipid nanoparticles of beeswax and carnauba wax. Iran. J. Chem. Chem. Eng..

[44] Ali A.S.M., Sarhan H.A., Magdy T. (2014). Preparation and characterization of phenytoin sodium niosomes for enhanced closure of skin injuries. Int. J. Pharm. Pharm. Sci..

[45] Aburahma M.H., Abdelbary G.A. (2012). Novel diphenyl dimethyl bicarboxylate provesicular powders with enhanced hepatocurative activity: Preparation, optimization, in vitro/in vivo evaluation. Int. J. Pharm..

[46] Abdelbary G., El-Gendy N. (2008). Niosome-encapsulated gentamicin for ophthalmic controlled delivery. AAPS PharmSciTech.

[47] Sguizzato M., Ferrara F., Hallan S.S., Baldisserotto A., Drechsler M., Malatesta M., Costanzo M., Cortesi R., Puglia C., Valacchi G. (2021). Ethosomes and transethosomes for mangiferin transdermal delivery. Antioxidants.

[48] Dey S.K., Mandal B., Bhowmik M., Ghosh L.K. (2009). Development and in vitro evaluation of Letrozole loaded biodegradable nanoparticles for breast cancer therapy. Braz. J. Pharm. Sci..

[49] Albash R., Abdelbary A.A., Refai H., El-Nabarawi M.A. (2019). Use of transethosomes for enhancing the transdermal delivery of olmesartan medoxomil: In vitro, ex vivo, and in vivo evaluation. Int. J. Nanomed..

[50] Badria F.A., Fayed H.A., Ibraheem A.K., State A.F., Mazyed E.A. (2020). Formulation of sodium valproate nanospanlastics as a promising approach for drug repurposing in the treatment of androgenic alopecia. Pharmaceutics.

[51] Alaaeldin E., Mostafa M., Mansour H.F., Soliman G.M. (2021). Spanlastics as an efficient delivery system for the enhancement of thymoquinone anticancer efficacy: Fabrication and cytotoxic studies against breast cancer cell lines. J. Drug Deliv. Sci. Technol..

[52] Mekkawy A.I., El-Mokhtar M.A., Nafady N.A., Yousef N., Hamad M.A., El-Shanawany S.M., Ibrahim E.H., Elsabahy M. (2017). In vitro and in vivo evaluation of biologically synthesized silver nanoparticles for topical applications: Effect of surface coating and loading into hydrogels. Int. J. Nanomed..

[53] Siddiqa A.J., Shrivastava N.K., Ali Mohsin M., Abidi M.H., Sharaf M.A.F., Shaikh T.A. (2021). In vitro release and degradation study of letrozole-loaded poly (lactic-co-glycolic acid) microparticles. JOM.

[54] Catauro M., Papale F., Bollino F., Piccolella S., Marciano S., Nocera P., Pacifico S. (2015). Silica/quercetin sol–gel hybrids as antioxidant dental implant materials. Sci. Technol. Adv. Mater..

[55] Mokale V.J., Patil H.I., Patil A.P., Shirude P.R., Naik J.B. (2016). Formulation and optimisation of famotidine proniosomes: An in vitro and ex vivo study. J. Exp. Nanosci..

[56] Maghsoodi M., Montazam S.h., Rezvantalab H., Jelvehgari M. (2020). Response surface methodology for optimization of process variables of atorvastatin suspension preparation by microprecipitation method using desirability function. Pharm. Sci..

[57] El-Sayed M.M., Hussein A.K., Sarhan H.A., Mansour H.F. (2017). Flurbiprofen-loaded niosomes-in-gel system improves the ocular bioavailability of flurbiprofen in the aqueous humor. Drug Dev. Ind. Pharm..

[58] Fathalla D., Youssef E.M., Soliman G.M. (2020). Liposomal and ethosomal gels for the topical delivery of anthralin: Preparation, comparative evaluation and clinical assessment in psoriatic patients. Pharmaceutics.

[59] Mazyed E.A., Zakaria S. (2019). Enhancement of dissolution characteristics of clopidogrel bisulphate by proniosomes. Int. J. Appl. Pharm..

[60] ElMeshad A.N., Mohsen A.M. (2016). Enhanced corneal permeation and antimycotic activity of itraconazole against Candida albicans via a novel nanosystem vesicle. Drug Deliv..

[61] Abdelrahman F.E., Elsayed I., Gad M.K., Elshafeey A.H., Mohamed M.I. (2017). Response surface optimization, Ex vivo and In vivo investigation of nasal spanlastics for bioavailability enhancement and brain targeting of risperidone. Int. J. Pharm..

[62] Gupta M., Vaidya B., Mishra N., Vyas S.P. (2011). Effect of surfactants on the characteristics of fluconazole niosomes for enhanced cutaneous delivery. Artif. Cells Blood Substit. Immobil. Biotechnol..

[63] Tabbakhian M., Tavakoli N., Jaafari M.R., Daneshamouz S. (2006). Enhancement of follicular delivery of finasteride by liposomes and niosomes 1. In vitro permeation and in vivo deposition studies using hamster flank and ear models. Int. J. Pharm..

[64] Alaaeldin E., Abou-Taleb H.A., Mohamad S.A., Elrehany M., Gaber S.S., Mansour H.F. (2021). Topical Nano-Vesicular Spanlastics of Celecoxib: Enhanced Anti-Inflammatory Effect and Down-Regulation of TNF-α, NF-κB and COX-2 in Complete Freund’s Adjuvant-Induced Arthritis Model in Rats. Int. J. Nanomed..

[65] Kanaani L., Javadi I., Ebrahimifar M., Ebrahimi Shahmabadi H., Akbarzadeh Khiyav A., Mehrdiba T. (2017). Effects of cisplatin-loaded niosomal nanoparticleson BT-20 human breast carcinoma cells. Asian Pac. J. Cancer Prev..

[66] Shaker D.S., Shaker M.A., Hanafy M.S. (2015). Cellular uptake, cytotoxicity and in-vivo evaluation of Tamoxifen citrate loaded niosomes. Int. J. Pharm..

[67] Fatemizadeh M., Tafvizi F., Shamsi F., Amiri S., Farajzadeh A., Akbarzadeh I. (2022). Apoptosis Induction, Cell Cycle Arrest and Anti-Cancer Potential of Tamoxifen-Curcumin Loaded Niosomes Against MCF-7 Cancer Cells. Iran. J. Pathol..

[68] Mehanna M.M., Sarieddine R., Alwattar J.K., Chouaib R., Gali-Muhtasib H. (2020). Anticancer activity of thymoquinone cubic phase nanoparticles against human breast cancer: Formulation, cytotoxicity and subcellular localization. Int. J. Nanomed..

[69] Choi E.J., Bae S.M., Ahn W.S. (2008). Antiproliferative effects of quercetin through cell cycle arrest and apoptosis in human breast cancer MDA-MB-453 cells. Arch. Pharm. Res..

[70] Li X., Zhou N., Wang J., Liu Z., Wang X., Zhang Q., Liu Q., Gao L., Wang R. (2018). Quercetin suppresses breast cancer stem cells (CD44+/CD24−) by inhibiting the PI3K/Akt/mTOR-signaling pathway. Life Sci..

[71] Niazvand F., Orazizadeh M., Khorsandi L., Abbaspour M., Mansouri E., Khodadadi A. (2019). Effects of quercetin-loaded nanoparticles on MCF-7 human breast cancer cells. Medicina.

[72] Azria D., Larbouret C., Cunat S., Ozsahin M., Gourgou S., Martineau P., Evans D.B., Romieu G., Pujol P., Pèlegrin A. (2005). Letrozole sensitizes breast cancer cells to ionizing radiation. Breast Cancer Res..

[73] Gibellini L., Pinti M., Nasi M., De Biasi S., Roat E., Bertoncelli L., Cossarizza A. (2010). Interfering with ROS metabolism in cancer cells: The potential role of quercetin. Cancers.

[74] Bourgonje A.R., Abdulle A.E., Al-Rawas A.M., Al-Maqbali M., Al-Saleh M., Enriquez M.B., Al-Siyabi S., Al-Hashmi K., Al-Lawati I., Bulthuis M.L. (2020). Systemic oxidative stress is increased in postmenopausal women and independently associates with homocysteine levels. Int. J. Mol. Sci..

[75] Doshi S., Agarwal A. (2013). The role of oxidative stress in menopause. J. Midlife Health.

[76] Lambert J.D., Elias R.J. (2010). The antioxidant and pro-oxidant activities of green tea polyphenols: A role in cancer prevention. Arch. Biochem. Biophys..

