# Supplementary material for: Combinatorial Therapy of Letrozole- and Quercetin-Loaded Spanlastics for Enhanced Cytotoxicity against MCF-7 Breast Cancer Cells
Source: Pharmaceutics. 2022 Aug 18;14(8):1727. doi: 10.3390/pharmaceutics14081727 (PMC9415400; doi:10.3390/pharmaceutics14081727)
Supplement: Supplementary file 1 [file pharmaceutics-14-01727-s001.zip › pharmaceutics-1825966-supplementary.pdf]

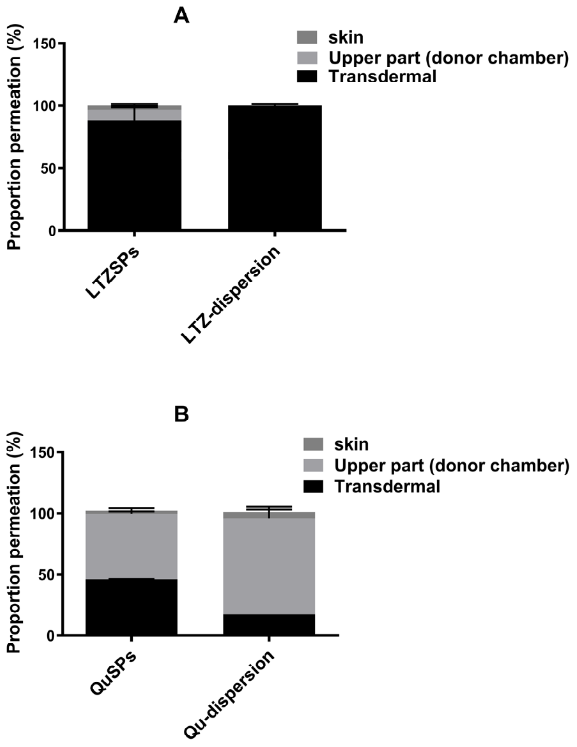

Figure S1. Proportions of the permeated amount of (**A**) LTZ from LTZSPs and LTZ-dispersion (**B**) Qu from QuSPs and Qu-dispersion through rat skin after 24-h incubation. Skin: stratum corneum, epidermis and dermis, while transdermal: receptor chamber. Abbreviations: LTZ; letrozole, Qu; Quercetin, LTZSPs; letrozole loaded spanlastics, QuSPs; Quercetin loaded spanlastics.

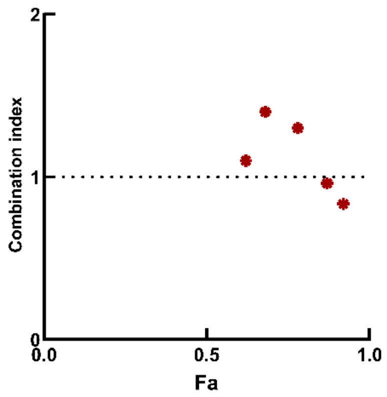

Figure S2. Combination index versus fraction affected plot for LTZSPs and QuSPs combination. Abbreviations: Fa; fraction affected, LTZSPs; letrozole-loaded spanlastics, QuSPs; quercetin-loaded spanlastics.

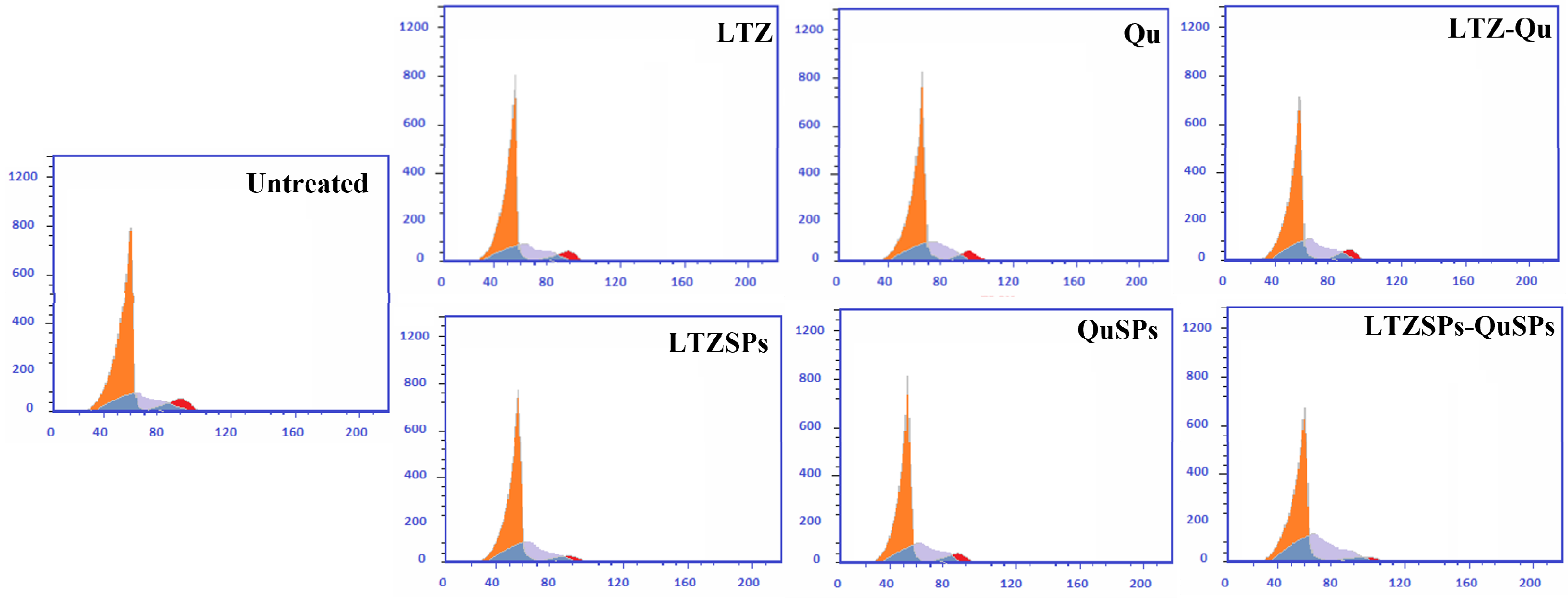

Figure S3. Cell cycle profile of MCF-7 cells treated with soluble LTZ, Qu, and their combination, LTZSPs, QuSPs and their combination.
